# Supplementary material for: Trends and Determinants of Cigarette Tax Increases in Japan: The Role of Revenue Targeting
Source: Int J Environ Res Public Health. 2022 Apr 18;19(8):4892. doi: 10.3390/ijerph19084892 (PMC9024842; doi:10.3390/ijerph19084892)
Supplement: Supplementary file 1 [file ijerph-19-04892-s001.zip › ijerph-1616078-supplementary.pdf]

Table S1. Searching for the minimum target of cigarette tax revenue.

Minimum Target of Cigarette Tax Revenue	Linear Probability Model (LPM)		Probit Model	
	Adjusted R^2		Log Likelihood	
	Model 1	Model 2	Model 1	Model 2
1.5	0.028	0.028	-21.246	-21.246
1.6	0.028	0.028	-21.246	-21.246
1.7	0.028	0.028	-21.246	-21.246
1.8	0.074	0.074	-19.904	-19.904
1.9	0.155	0.109	-18.468	-18.468
2.0	0.204	0.280	-17.755	-15.296
2.1	0.008	0.193	-21.077	-17.915
2.2	0.044	0.123	-20.325	-19.102
2.3	0.009	0.116	-20.922	-19.168
2.4	0.028	0.116	-21.246	-19.158
2.5	0.028	0.116	-21.246	-19.158
N	36	36	36	36

Text S1. Time series analysis of cigarette tax revenue and tax rate

1. Objective and methods

The objective of this supplemental analysis was to examine whether an increase in cigarette tax was followed by a reduction in cigarette tax revenue. Using a vector auto-regression (VAR) model, we tested Granger causality and analyzed the impulse response function[1, 2] to establish the relationship between the time series of expected tax revenue and tax rate.

2. Data

We used the following data (same data as in the main text).

a. Total pieces of cigarettes sold (*Vol*), 1985-2021. Source: Tobacco Institute of Japan

b. Total cigarette tax revenue in JPY (*TAX*), 1985-2021. Source: Ministry of Finance and Ministry of Internal Affairs and Communications of Japan

Following the main text, we generated two variables for analysis.

I. Cigarette tax per piece $PTAX_t = TAX_t / Vol_t$

II. Expected tax revenue from the previous year at a pace average over the last five years $ETAX_t = PTAX_{t-1}(Vol_{t-1}/Vol_{t-6})^{1/5} Vol_{t-1}$

Note that $ETAX_t$ is equivalent to $E(V_t)$ in the main text.

3. Result

Testing for unit root and cointegration

We transformed $PTAX_t$ and $ETAX_t$ into natural logarithmic form and conducted the analyses. First, we tested unit root of the time series using Augmented Dicky-Fuller test[1] and Kwiatkowski-Phillips-Schmidt-Shin test.[3] As the result, we found that both variables follow a unit root process. Next, we proceeded to analyze cointegration between the two variables, and a Johansen test rejected cointegration.[2]

Granger causality

We tested Granger causality using a vector autocorrelation (VAR) model of $lnPTAX_t$ and $lnETAX_t$. The optimal lag in the model was determined by Akaike Information Criterion. We adopted an approach developed by Toda and Yamamoto[4] with a constant term and linear trend (Model 1), as well as a standard first-difference model with a constant term (Model 2).

We found, for Model 1 and 2, $ETAX_t$ Granger-causes $PTAX_t$ at significance level of 1%, meaning that a change in tax rate is explained by a preceding change in expected tax revenue. We also found that, for Model 1 and 2, $PTAX_t$ Granger-causes $ETAX_t$ at significance level of 1%, meaning that a change in expected tax revenue is explained by a preceding change in tax rate. This is consistent with a stylized prediction that a tax hike increases revenue at least in the short run.

Impulse response function

Given we confirmed Granger causality from $ETAX_t$ to $PTAX_t$, we examined the direction of a change in $PTAX_t$ following a standard-deviation (SD) point change in $ETAX_t$, analyzing an impulse response function.[1,2] As a result, we found, for Model 1 and 2, a shock on $ETAX_t$ by a SD point would significantly decrease $PTAX_t$ by 0.07 to 0.40% after one or two years. This means that a SD point decline in expected tax revenue would lead to an increase in tax rate.

4. Remarks

Our analysis of Granger causality confirmed that a change in expected tax revenue predetermines a change in tax rate. We also showed that a reduction of expected tax revenue would lead to an increase in tax rate.

References

36. Hamilton, J.D. *Time Series Analysis*; Princeton University Press: Princeton, NJ, USA; 1994.
37. Lütkepohl, H. *New Introduction to Multiple Time Series Analysis*; Springer: New York, NY, USA; 2005.
38. Kwiatkowski, D.; Phillips P.C.P.; Schmidt, P.;Shin, Y. Testing the null hypothesis of stationarity against the alternative of a unit root: How sure are we that economic time series have a unit root? *J. Econom.* **1992**, *54*, 159–178.
39. Toda, H.Y.; Yamamoto, T. Statistical inference in vector autoregressions with possibly integrated process. *J. Econom.* **1995**, *66*, 225–250.
